# Unraveling the protective role of sulfide signaling in plants against climate-induced abiotic stress

**DOI:** 10.1093/jxb/eraf527

**Published:** 2025-11-28

**Authors:** Angeles Aroca, Reyes Carrillo, David Montesinos-Pereira, Carmen Luque, Luis C Romero, Cecilia Gotor

**Affiliations:** Instituto de Bioquímica Vegetal y Fotosíntesis, Consejo Superior de Investigaciones Científicas and Universidad de Sevilla, Avenida Américo Vespucio, 49, Seville 41092, Spain; Instituto de Bioquímica Vegetal y Fotosíntesis, Consejo Superior de Investigaciones Científicas and Universidad de Sevilla, Avenida Américo Vespucio, 49, Seville 41092, Spain; Instituto de Bioquímica Vegetal y Fotosíntesis, Consejo Superior de Investigaciones Científicas and Universidad de Sevilla, Avenida Américo Vespucio, 49, Seville 41092, Spain; Instituto de Bioquímica Vegetal y Fotosíntesis, Consejo Superior de Investigaciones Científicas and Universidad de Sevilla, Avenida Américo Vespucio, 49, Seville 41092, Spain; Instituto de Bioquímica Vegetal y Fotosíntesis, Consejo Superior de Investigaciones Científicas and Universidad de Sevilla, Avenida Américo Vespucio, 49, Seville 41092, Spain; Instituto de Bioquímica Vegetal y Fotosíntesis, Consejo Superior de Investigaciones Científicas and Universidad de Sevilla, Avenida Américo Vespucio, 49, Seville 41092, Spain; Universität Heidelberg, Germany

**Keywords:** Drought, high carbon dioxide, hydrogen sulfide, persulfidation, salinity, signaling, stress responses, temperature

## Abstract

Hydrogen sulfide (H_2_S) is now widely recognized as a signaling molecule playing a crucial role as a regulator of essential biological processes. This review outlines current knowledge of the endogenous H_2_S sources in different cellular compartments and highlights its principal mechanisms of action. In the current context of climate change, the involvement of H_2_S in plant stress responses has gained increasing attention. H_2_S has been shown to alleviate the adverse effects of various abiotic stresses by regulating specific physiological and molecular responses, enabling stress tolerance and crop resilience. A comprehensive overview is provided on how H_2_S regulates adaptive responses to environmental stressors, such as elevated atmospheric carbon dioxide, drought, soil salinization, and thermal stress. Notably, protein persulfidation has emerged as the main mechanism through which H_2_S exerts these regulatory effects.

## Teaser

This review underscores the key role of H_2_S in enhancing plant resilience against abiotic stress prevalent in the current climate change scenario. Endogenous sources of H_2_S and its mechanisms of action are also discussed.

## Introduction

The sustainability and resilience of global agroecosystems are increasingly compromised by the multifaceted impacts of climate change, which include a rising atmospheric carbon dioxide (CO_2_) concentration, concurrent increases in global temperature, altered precipitation patterns leading to more frequent and intense droughts, and the consequent expansion of soil salinization. These environmental perturbations are causing a significant and increasing threat to worldwide agricultural productivity (Fig. 1). These climatic stressors directly modulate fundamental phenotypic and biochemical processes in plant physiology, affecting its development and crop yield.

**Fig. 1.**
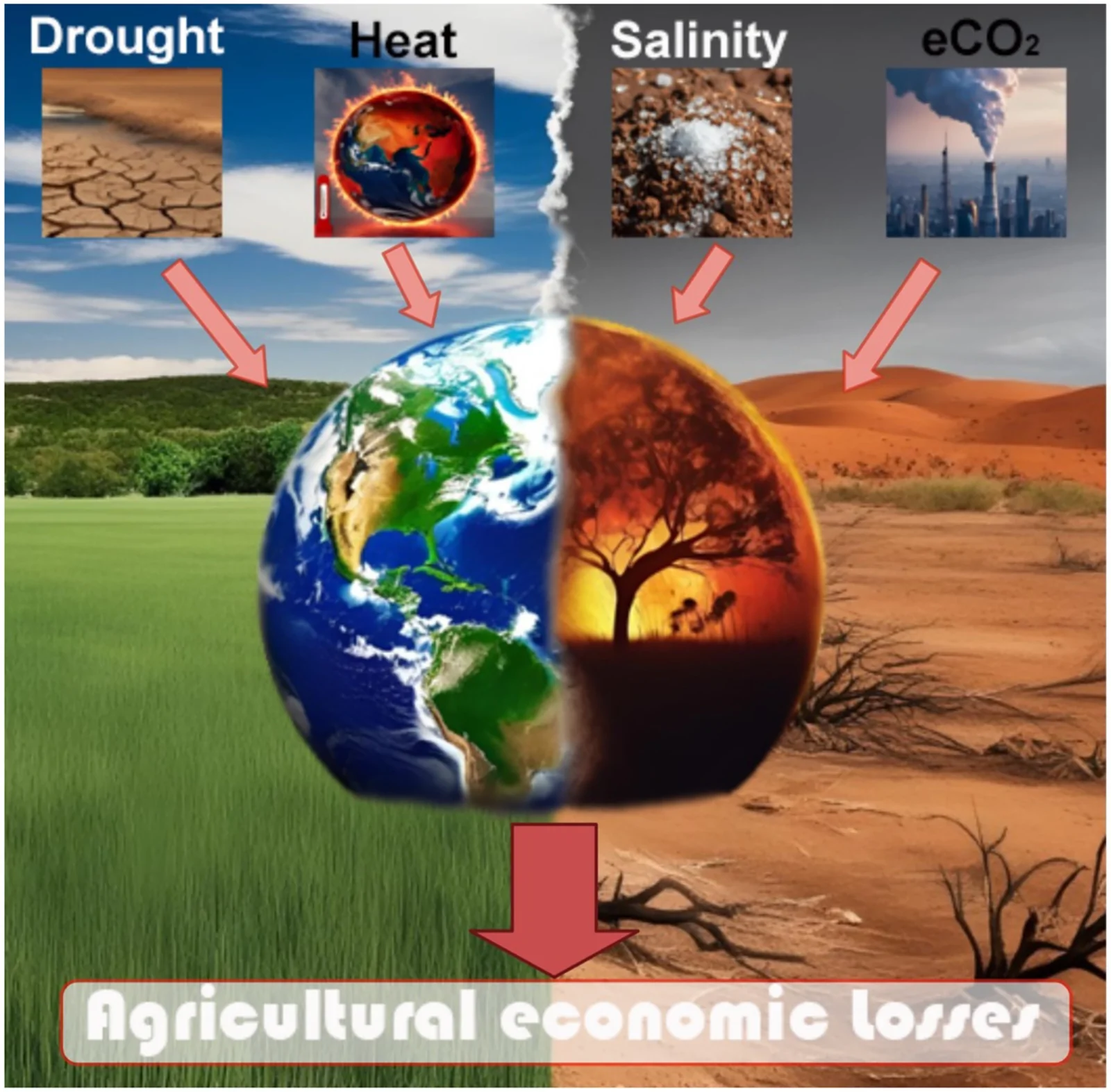
Major abiotic stressors affecting crop productivity under climate change scenarios. Drought, heat stress, soil salinity, and elevated atmospheric CO_2_ (eCO_2_) are key environmental factors that negatively impact plant growth and yield, contributing to significant agricultural economic losses. Background generated with Canva AI software and amended in Photoshop.

The progressive rise in atmospheric CO_2_ concentration, which currently exceeds 415 ppm (NOAA, 2024), elicits a complex suite of physiological responses in terrestrial vegetation. Although plants subjected to elevated CO_2_ concentrations may exhibit an initial increase in growth, potentially increasing biomass accumulation, this CO_2_ fertilization effect is frequently contingent upon other environmental factors and can be associated with diminished foliar nutrient concentrations (Taub *et al*., 2008). In fact, substantial evidence indicates that photorespiration plays a critical role in essential metabolic processes, including nitrogen and sulfur assimilation (Bloom *et al*., 2002; Abadie and Tcherkez, 2019). Therefore, the disruption of photorespiration by elevated CO_2_ concentrations can compromise crop productivity and nutritional quality by diminishing protein synthesis in harvested tissues (Wattal and Siddiqui, 2015; Aroca *et al*., 2023; García-Calderón *et al*., 2023; Ekele *et al*., 2025). Furthermore, alterations in stomatal conductance and water use efficiency exhibit species-specific variability, with implications for plant water dynamics under future climate scenarios (Leakey *et al*., 2009). The statistically significant increase in global mean surface temperature, with recent decadal analyses indicating unprecedented warming (IPCC, 2024), imposes heat stress on cultivated plant species, which can induce protein denaturation, impairment of photosynthetic efficiency, accelerated phenological progression leading to truncated reproductive phases, and elevated rates of respiration, collectively resulting in yield attenuation (Craufurd and Wheeler, 2009).

Besides, altered precipitation patterns are linked to a higher incidence and severity of drought events across numerous agriculturally significant regions (Sheffield *et al*., 2012). Water deficit directly constrains plant growth and development via osmotic stress, reduced nutrient mobilization, and compromised photosynthetic activity, leading to substantial yield reductions and heightened probabilities of complete crop failure.

The gradual expansion of soil salinization, often exacerbated by diminished freshwater availability and increased evapotranspiration in arid and semi-arid environments, degrades arable land quality. Elevated concentrations of soluble salts within the root zone impede water uptake, induce ion toxicity, and disrupt essential nutrient acquisition, severely limiting crop productivity and potentially rendering agricultural land unusable (Rengasamy, 2010).

Quantitative analyses have consistently demonstrated the negative impacts of these climate change drivers on global crop yields, leading to big economic losses worldwide (Table 1). The effect of these yield reductions presents a substantive challenge to global food security and the economic stability of agricultural communities. The various agricultural systems exhibit differential sensitivities to climate change and its associated abiotic stresses (Table 2).

**Table 1.** Global climate-driven agricultural losses in 2024 by continent

Continent	Key affected crops	Estimated loss (billion US$)	Main climate drivers	Sources
Africa	Maize, sorghum, coffee	7–12	Drought	AGRA (2024); FEWS NET (2024)
Asia	Rice, wheat, palm oil	15–25	Heatwaves, floods, cyclones	FAO (2024a, 2025)
Europe	Wheat, olives, grapes	8–15	Drought, extreme heat	European Commission Joint Research Centre (2024)
North America	Corn, soybeans, wheat	10–20	Floods, drought, wildfires	CONAB (2024); NASS (2024)
South America	Soybeans, coffee, corn	12–18	Floods, landslides	CONAB (2024); FAO *et al*. (2025)
Oceania	Wheat, sugarcane	2–5	Drought, cyclones	ABARES (2024 , 2025)

**Table 2.** Crop yield losses and economic costs by climate stressor (2024 estimates as global averages with regional hotspots highlighted)

Stressor	Yield loss and main crop affected	2024 economic loss (billion US$)	Worst-hit regions	Reference
Heatwaves	6–18% (wheat)	28–45	South Asia, Africa	Asseng *et al*. (2015); UNDRR (2024)
Drought	12–35% (maize)	32–55	Mediterranean, Brazil	European Commission Joint Research Centre (2024); FAO (2024b)
Flooding	18–28% (rice)	22–40	South-east Asia, US Midwest	Li *et al*. (2025)
CO_2_ effects	7% nutrient density (wheat and rice)	12–22 (mainly nutritional quality losses)	Global (variable)	Myers *et al*. (2014); Ekele *et al*. (2025)
Salinization	25–60% (vegetatables and grains)	9–18	Nile Delta, Bangladesh	IPCC (2022, 2024)

The scientific evidence clearly establishes that climate change will harm agricultural productivity, making a full understanding of the biophysical processes and the development of adaptive management practices a priority. The drivers of climate change collectively exert serious abiotic stress on agricultural crops, disrupting fundamental metabolic pathways and threatening crop yield. A key consequence of these abiotic stresses is the overproduction of reactive oxygen species (ROS), which alter cellular redox homeostasis, inhibiting photosynthetic efficiency and impairing enzyme function. The accumulating consequences of climate change will make it increasingly challenging for future agricultural systems to cope without incorporating climate change mitigation measures (Wahid *et al*., 2007).

One emerging area of research highlights hydrogen sulfide (H_2_S) as a gaseous signaling molecule with an important role in plant responses to heat, drought, and salinity stress (Aroca *et al*., 2020, 2021). Exogenous H_2_S application has also been shown to affect systems of antioxidant defense, stomatal regulation, and photosynthetic potential that would modulate the positive effects of abiotic stress-resistant metabolism while enhancing crop yield in challenging environments (Aroca *et al*., 2021; Khanna *et al*., 2021; Wang *et al*., 2024).

This article aims to provide a comprehensive overview of current knowledge regarding the role of H_2_S as a signaling molecule and regulator of ROS metabolism, emphasizing its potential as a key factor in mitigating climate-induced abiotic stresses in agricultural crops. We seek to highlight the promising role of H_2_S to enhance crop resilience in the face of a threatening changing global climate.

## Hydrogen sulfide in plants

Although H_2_S is traditionally recognized as a toxic compound, it is now well established as a signaling molecule below a certain concentration threshold, comparable to the physiological endogenous concentration (as described below), similar to other molecules such as carbon monoxide (CO), nitric oxide (NO), and hydrogen peroxide (H_2_O_2_). Since the initial identification of H_2_S as an endogenous neuromodulator (Abe and Kimura, 1996), extensive research in both plant and animal systems has positioned H_2_S signaling as a prominent topic due to its significant biomedical and biotechnological applications.

H_2_S plays a crucial role in regulating a wide variety of essential processes in mammals, and it is clinically relevant because alterations in its metabolism are linked to various pathologies (Zivanovic *et al*., 2019; Giovinazzo *et al*., 2021; Petrovic *et al*., 2021; Paul, 2022). Similarly, H_2_S has a vital role as a regulator of essential biological processes in plants, such as developmental programs including germination, root development, leaf senescence, and postharvest and fruit ripening. H_2_S also represses the progression of autophagy, delays programmed cell death, and regulates photosynthesis and stomatal movement, thus being a universal regulator critical for plant performance (Aroca *et al*., 2018, 2020; Huo *et al*., 2018; Ziogas *et al*., 2018; Gotor *et al*., 2019; Pantaleno *et al*., 2021; Aroca and Gotor, 2022; Huang and Xie, 2023; Jaiswal *et al*., 2025).

In the current context of climate change, the role of H_2_S in plant stress responses has gained increasing attention. H_2_S has been shown to mitigate the adverse effects of various abiotic stresses by regulating specific physiological and molecular responses. Studies across diverse plant species have demonstrated its involvement in responses to elevated CO_2_, drought, salinity, heat, hypoxia, heavy metal exposure, and other environmental challenges (Aroca *et al*., 2021, 2023; Zhang *et al*., 2021; Mingjian *et al*., 2025; Yu *et al*., 2025b). The mechanism by which H_2_S enhances plant resilience to these stresses will be described in the following sections.

## Hydrogen sulfide biosynthetic pathways in different plant cellular compartments

In both mammals and plants, H_2_S is produced endogenously by cells through the functions of different enzymes involved in cysteine metabolism. However, in plants, the scenario is more complex due to the dual nature of H_2_S as a nutrient and a regulator. Thus, as a nutrient, H_2_S is produced during the photosynthetic sulfate assimilation pathway located in the chloroplast, by the action of the enzyme sulfite reductase, which reduces sulfite to H_2_S (Takahashi *et al*., 2011). In this pathway, H_2_S is an intermediate for the biosynthesis of the first reduced sulfur metabolite, cysteine, which is a precursor of the majority of sulfur-containing metabolites of the plant cell (Romero *et al*., 2014; Gotor *et al*., 2017).

When the fate of H_2_S is cell signaling, different sources are involved, and the contributions of various enzymes have been described, without disregarding the H_2_S production through reductive sulfate assimilation in the chloroplast (Gotor *et al*., 2019). As a matter of fact, the generation of H_2_S is closely linked to the biosynthesis of cysteine catalyzed by *O*-acetylserine(thiol) lyases (OASTLs), which is the last step of the photosynthetic sulfate assimilation pathway. These OASTLs are located in chloroplasts, mitochondria, and the cytosol, although the cytosol predominantly contributes to the biosynthesis of cysteine (Takahashi *et al*., 2011). Therefore, whereas chloroplasts are the main source of H_2_S from the assimilatory pathway, with an endogenous concentration of >120 µM having been estimated in Arabidopsis, H_2_S generated from cysteine constitutes the majority produced in the cytosol, with an assessed concentration of >50 µM (Krueger *et al*., 2009) (Fig. 2).

**Fig. 2.**
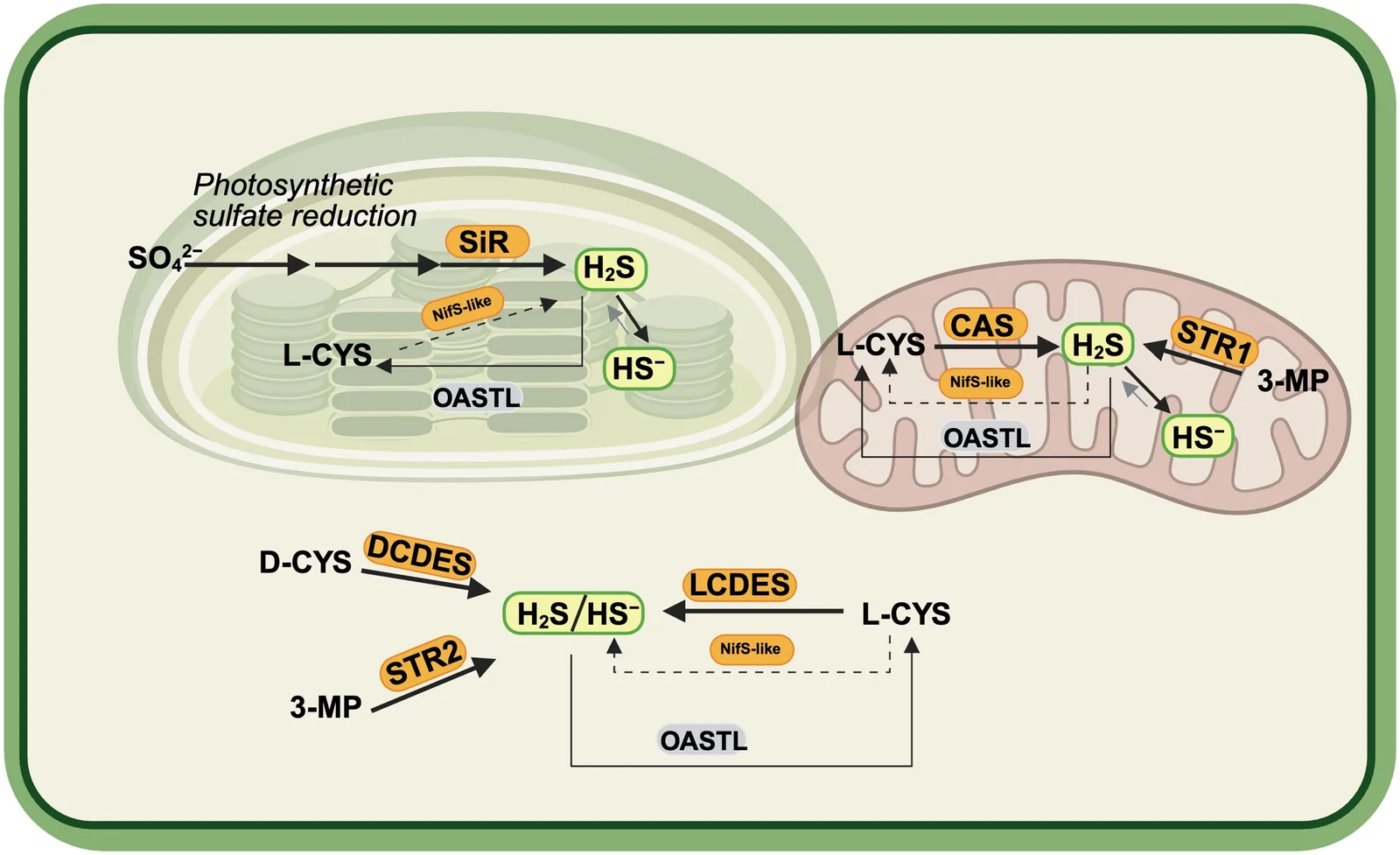
Endogenous production of hydrogen sulfide (H_2_S) in Arabidopsis cells. H_2_S is produced in different cell compartments through the action of various enzymatic sources. CAS, β-cyanoalanine synthase; DCDES, D-cysteine desulfhydrase; LCDES, L-cysteine desulfhydrase; SiR, sulfite reductase; STR1 and STR2, sulfurtransferase located in mitochondria and cytosol, respectively. NifS-like, L-cysteine desulfurase, has been proposed, but not yet demonstrated. D-CYS, D-cysteine; L-CYS, L-cysteine; HS^–^, hydrosulfide anion; 3-MP, 3-mercaptopyruvate; SO_4_^2–^, sulfate. Created in BioRender. Aroca, A. (2025) https://BioRender.com/tj2it1m.

The enzymatic production of H_2_S from cysteine takes place through the action of various types of cysteine-degrading enzymes (Gotor *et al*., 2019; Hilal *et al*., 2023). In the cytosol, the L-cysteine desulfhydrases (LCDESs) catalyze the desulfuration of L-cysteine to produce H_2_S, ammonia, and pyruvate; in particular, one LCDES from Arabidopsis, named DES1, has been characterized in detail (Álvarez *et al*., 2010). Deficiency of DES1 reduces the total endogenous H_2_S concentration by 20–30% in leaves and strongly affects plant physiology (Álvarez *et al*., 2012a). The *des1* mutant has become a key molecular tool that is extensively used to demonstrate the essential role of DES1-generated H_2_S in regulating numerous biological processes, including autophagy (Aroca and Gotor, 2022; Tang and Bassham, 2022) and stomatal movement (Aroca *et al*., 2021; Pantaleno *et al*., 2021; Siodmak and Hirt, 2021). Similar to LCDES, D-cysteine desulfhydrase (DCDES) generates H_2_S from D-cysteine (Papenbrock *et al*., 2007), and a significant role for this enzyme in the regulation of different biological processes has been implicated (Xiang *et al*., 2023; Zhao *et al*., 2023). Besides the cytosol, mitochondria are another source compartment of H_2_S metabolically generated from cysteine by the action of β-cyanoalanine synthase during cyanide detoxification (Álvarez *et al*., 2012b).

Additional enzymes that have been proposed to potentially produce sulfur from cysteine are the L-cysteine desulfurases, known as NifS-like proteins. These proteins participate in the formation of iron–sulfur clusters, thiamine, biotin, lipoic acid, and molybdenum cofactor, and are localized in the cytosol, chloroplast, and mitochondria (Van Hoewyk *et al*., 2008; Caubriere *et al*., 2023; Pedroletti *et al*., 2023 ). Similar to cysteine desulfurases, the sulfurtransferases (STRs) generate sulfur from cysteine indirectly after its transformation to 3-mercaptopyruvate, and both enzymes are transiently persulfidated in their reaction cycle. STRs are characterized by the presence of at least one rhodanese homology domain, which usually contains the catalytic, persulfidated cysteine. Thus, it has been suggested that STRs are involved in sulfur trafficking through *trans*-persulfidation when the persulfide group formed on the catalytic cysteine is transferred to nucleophilic acceptors such as glutathione, proteins, or small metabolites. Additionally, some STRs may be involved in H_2_S biogenesis when persulfidated STRs react with thioredoxin (TRX) or glutaredoxin (GRX) (Selles *et al*., 2019). In Arabidopsis, the isoforms STR1 and STR2, located in mitochondria and cytosol, respectively, have been biochemically characterized as 3-mercaptopyruvate sulfurtransferases, and their role in the generation of H_2_S and glutathione persulfide was emphasized (Moseler *et al*., 2021) (Fig. 2).

The H_2_S generation located in different cell compartments could be due to the chemical properties of this molecule. H_2_S is a weak acid, with p*K*_a1_ and p*K*_a2_ values of 6.9 and >12, respectively, and it dissociates into the anionic forms HS^−^ and S^2−^ (Kabil and Banerjee, 2010 ). In aqueous solution, H_2_S exists as a neutral form together with the anionic HS^−^ form; this anionic form constitutes two-thirds of the total H_2_S content under physiological pH conditions. Therefore, the distribution of H_2_S between compartments is restricted only to the neutral form of H_2_S due to its lipid solubility in cell membranes, which the anionic forms are unable to permeate (Cuevasanta *et al*., 2012). This characteristic is more pronounced in the chloroplast stroma under optimal conditions for photosynthetic reactions, and in the metabolically active mitochondria stroma, which have a relatively basic pH of 8 (Shen *et al*., 2013). Another aspect that we cannot exclude as a reason for the different locations of H_2_S generation is the specificity of each variety of H_2_S enzymatic sources for certain biological processes regulated by this molecule. In this context, these sources would be activated under specific physiological conditions or timing.

## Hydrogen sulfide signaling mechanisms

The main physiological characteristics of H_2_S are based on its chemical properties, mainly nucleophilicity and reducing power (Mustafa *et al*., 2009; Kabil and Banerjee, 2010). Based on these properties, initially, three possible mechanisms of action were proposed (Fig. 3). The best studied is the reaction with the thiol group of cysteine residues in proteins, leading to the formation of corresponding persulfides (−SSH), which results in a post-translational modification known as persulfidation. The other less-researched mechanisms of action of H_2_S are the binding to metal centers of metalloproteins and the interaction with ROS and reactive nitrogen species (RNS), thus acting as an indirect antioxidant. However, after substantial research in recent years, as described below, the consensus conclusion is that persulfidation is the main mechanism of H_2_S action, and that the other two proposed mechanisms—binding to metalloproteins and reducing the oxidative state—seem to be outcomes of the persulfidation of specific proteins.

**Fig. 3.**
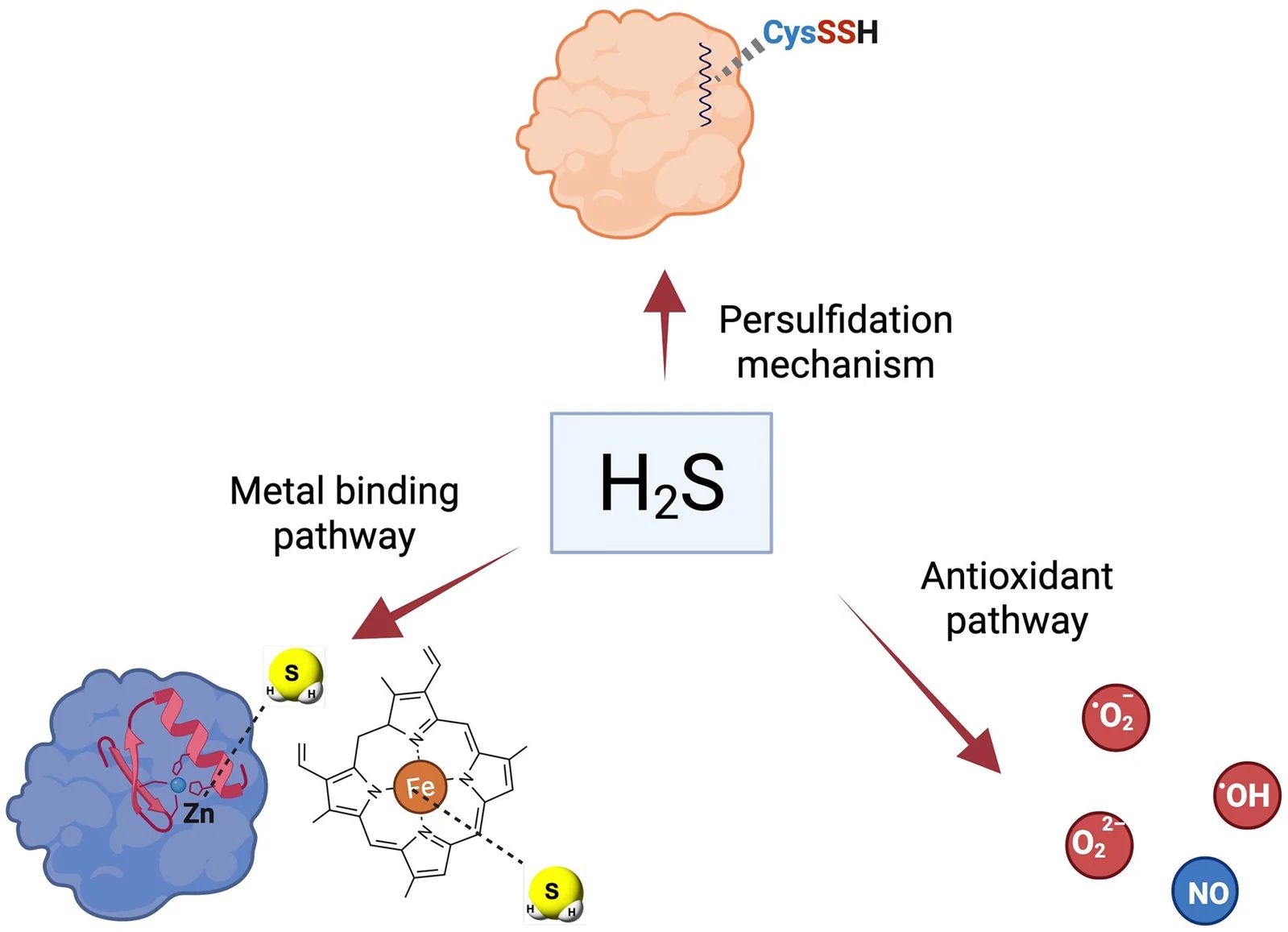
Hydrogen sulfide (H_2_S) signaling pathways in plants. The diagram highlights three initially proposed mechanisms by which H_2_S mediates its biological effects: (i) protein persulfidation, (ii) metal binding interactions involving zinc (Zn) and iron (Fe), and (iii) reducing oxidative stress by targeting reactive oxygen and nitrogen species, including superoxide radical (O_2_•⁻), peroxide ion (O_2_^2^⁻), hydroxyl radical (•OH), and nitric oxide (NO). Created in BioRender. Romero, L. (2026) https://BioRender.com/mehykp9.

### Persulfidation

This oxidative modification of cysteine residues to form persulfides leads to alterations in protein functions, affecting catalytic activity, subcellular localization, and protein–protein interactions, among other processes. These changes lead to significant physiological effects (Gotor *et al*., 2019, 2022; Aroca *et al*., 2021; Huang and Xie, 2023). Since the first report of plant persulfidation, in Arabidopsis (Aroca *et al*., 2015), several high-throughput proteomic analyses in Arabidopsis and various crop species have revealed that persulfidation is a widespread post-translational modification in plant systems. Currently, an extensive catalog of persulfidation-susceptible proteins is available for Arabidopsis (Aroca *et al*., 2017; Laureano-Marin *et al*., 2020; Jurado-Flores *et al*., 2021, 2023a, 2023b; García-Calderón *et al*., 2023; De Brasi-Velasco *et al*., 2025), rice (Zhang *et al*., 2024), pepper (Muñoz-Vargas *et al*., 2024), and bean (Matamoros *et al*., 2024), comprising a total of 11 700 proteins. A recent review (Romero *et al*., 2025) provides a comprehensive overview of this modification.

Although substantial data exist, the precise mechanism underlying protein persulfidation and the identity of the pesulfidating species remain subjects of debate. As noted, H_2_S is a reductant, and H_2_S or HS^−^ cannot directly react with protein thiols, where the sulfur is in the same oxidation state. Therefore, H_2_S has been proposed to react with oxidized cysteine residues and, based on thermodynamic studies, the reaction with sulfenylated residues (−SOH) seems to be the most favorable process (Cuevasanta *et al*., 2015). This mechanism implies a significant crosstalk between H_2_O_2_ and H_2_S signaling pathways, where protein persulfidation relies on prior sulfenylation of protein. This temporal dynamic has been shown in various biological systems (Zivanovic *et al*., 2019; García-Calderón *et al*., 2023). Notably, a comparative study revealed that 82% of sulfenylated proteins in Arabidopsis are also persulfidated (Aroca *et al*., 2021).

Another interesting aspect of persulfidation is its protective role against cysteine overoxidation and, consequently, against protein degradation. Sulfenylated residues can be reversed back to thiols by the action of biological reductant agents, such as the TRX systems. However, persistent oxidation provokes further oxidation of sulfenylated residues to generate sulfinic (−SO_2_H) or sulfonic (−SO_3_H) motifs that are mainly irreversible modifications. In contrast, oxidation of persulfidated groups generates perthiosulfenic (−SSOH), perthiosulfinic (−SSO_2_H), or perthiosulfonic (−SSO_3_H) groups, which can be reduced back to thiol by the action of reductants (Filipovic *et al*., 2018; Gotor *et al*., 2019). In this sense, the reversing of persulfidation was demonstrated in animal systems by the TRX systems, thus proving the function of TRX as a depersulfidase (Wedmann *et al*., 2016; Zivanovic *et al*., 2019). A recent study in the mitochondrial TRX*o*1 from Arabidopsis established the ability of this thioredoxin to function as a depersulfidase (De Brasi-Velasco *et al*., 2025), suggesting a conserved mechanism for fine-tuning H_2_S signaling and maintaining redox homeostasis across kingdoms.

Other mechanisms contributing to protein persulfidation include transsulfuration, which consists of the transfer of a sulfur atom from a persulfide donor to a thiol-containing substrate, catalyzed by sulfurtransferases such as the 3-mercaptopyruvate sulfurtransferase, which thus act as a persulfidase (Filipovic *et al*., 2018). In Arabidopsis, STR1 and STR2 are the 3-mercaptopyruvate sulfurtransferases that are better studied (Moseler *et al*., 2021). Additionally, low-molecular-weight persulfides such as cysteine and glutathione persulfides may serve as sulfur donors (Vignane and Filipovic, 2023), although their presence in plants has not yet been confirmed.

### Binding to metal centers

Based on the reactivity of H_2_S with metal ions, it has been proposed that H_2_S can covalently bind to the metal center of metalloproteins, representing an additional mechanism of action (Filipovic *et al*., 2018). A well-characterized example is the interaction of H_2_S with the heme porphyrins of the cytochrome *c* oxidase, which at high concentrations of H_2_S leads to inhibition of the mitochondrial respiratory chain (Birke *et al*., 2015). In mammals, this process induces the release of cytochrome *c* during apoptosis and stimulates the persulfidation of procaspase 9 (Vitvitsky *et al*., 2018). Other metalloproteins that can be targets of H_2_S are heme-containing proteins such as leghemoglobin (Boubeta *et al*., 2020) and Zn-finger proteins (Lange *et al*., 2019).

Notably, Zn-finger proteins have emerged as elements playing an essential role in a non-enzymatic mechanism of protein persulfidation. Proteomic analyses across various biological systems have revealed a high proportion of persulfidated Zn-finger proteins (H. Li *et al*., 2024). The cysteine residues coordinated to the Zn ions can be prone to persulfidation, altering the conformation of the Zn-finger motif and therefore its interaction with DNA, RNA, or proteins. Similarly, other metal ions may influence the persulfidation of proximal cysteines. In plant proteomic studies, metalloproteins are consistently overrepresented among persulfidated targets, such as Zn-finger motifs or metal cofactors like heme iron, copper, or iron–sulfur clusters (Romero *et al*., 2025), suggesting a broader role for metal coordination in regulating this post-translational modification.

### Reducing oxidative stress

Given that H_2_S is a reductant molecule, alternative mechanisms by which it can function as a regulator include its potential direct interaction with biological oxidants such as various ROS and RNS (Filipovic *et al*., 2012; Zaffagnini *et al*., 2019). Through such interactions, H_2_S could modulate the level of these oxidants, thereby influencing the cellular redox state. However, direct reactions with ROS and RNS have not been demonstrated, and it appears that the impact of H_2_S on cellular redox homeostasis is indirect, either through the reaction of oxidized cysteines in proteins, preventing protein overoxidation, or by activating antioxidant systems.

Under stress conditions, plants typically overproduce ROS, leading to oxidative damage (Miller *et al*., 2010). Numerous studies have shown that both exogenous and endogenous H_2_S significantly reduce ROS accumulation in plants (Zhou *et al*., 2020; Bu *et al*., 2024; Song *et al*., 2024; Zhang *et al*., 2024; Alvi *et al*., 2024b; Carrillo *et al*., 2025; Yu *et al*., 2025a). These findings consistently demonstrate that H_2_S alleviates oxidative stress by enhancing plants’ antioxidant capacity.

Proteomic analysis of persulfidated proteins in plants has revealed a significant overrepresentation of proteins and enzymes involved in redox homeostasis (Romero *et al*., 2025 ). This group includes key antioxidant enzymes such as peroxidases, catalases (CATs), superoxide dismutases (SODs), methionine sulfoxide reductases, and thioredoxins. The functional consequences of persulfidation for these enzymes have been extensively studied. For example, the persulfidation of ascorbate peroxidases (APXs) in Arabidopsis and tomato induces their enzymatic activities (Aroca *et al*., 2015; Li *et al*., 2020; De Brasi-Velasco *et al*., 2025), although in bean they remain unaffected (Matamoros *et al*., 2024). In Arabidopsis, persulfidation of dehydroascorbate reductase and CAT increases their enzymatic activity (García-Calderón *et al*., 2023; De Brasi-Velasco *et al*., 2025), whereas in tomato the opposite effect is observed, with CAT being inhibited (Li *et al*., 2020).

In summary, H_2_S effectively mitigates oxidative stress under various environmental stress conditions. The persulfidation of proteins involved in maintaining redox balance appears to be a key mechanism by which H_2_S exerts its antioxidant effects. Thus, persulfidation emerges as a central determinant of the antioxidant capacity of H_2_S in plant systems.

## Hydrogen sulfide signaling in responses to abiotic stress induced by climate change

As previously discussed, under the current climate change conditions, H_2_S has gained prominence as a crucial factor in plant protection and the mitigation of environmental stress. A growing body of research in recent years has highlighted its multifaceted role in enhancing plant resilience. The following sections examine a range of studies that have identified key H_2_S-regulated physiological processes that contribute to improved tolerance of adverse conditions with the greatest impact on crop productivity.

### High carbon dioxide

Climate change is causing a considerable increase in atmospheric CO_2_ concentration, driving fundamental changes associated with both plant physiology and metabolism. In C_3_ species, the rise in CO_2_ availability leads to a suppression of photorespiration by favoring the carboxylase activity of the Rubisco enzyme over its oxygenase activity (Xu *et al*., 2015). Although this increases carbon assimilation through photosynthesis, it also suppresses the regulatory functions performed by photorespiration. Consequently, plants may suffer oxidative stress, deficiencies in nitrogen assimilation, and alterations in ATP synthesis (Betti *et al*., 2016).

A major consequence of this suppression is the accumulation of ROS that alter cellular equilibrium and condition plant performance. Previous studies have demonstrated that Arabidopsis plants grown under non-photorespiratory conditions (NPC; 0.7% CO_2_) accumulated high levels of H_2_O_2_, which contributes to oxidative stress and stomatal closure (García-Calderón *et al*., 2023). Additionally, treatment of plants growing under these conditions with exogenous H_2_S can markedly reduce H_2_O_2_ concentrations in leaves and promote stomatal reopening, which highlights the signaling role of H_2_S in mitigating oxidative damage (Aroca *et al*., 2020). This ROS reduction is related to H_2_S-mediated modulation of key redox enzymes such as CAT, responsible for H_2_O_2_ removal in peroxisomes, whose activity increases after H_2_S application under elevated CO_2_ concentrations (García-Calderón *et al*., 2023).

As previously mentioned, H_2_S signaling primarily occurs through protein persulfidation. In fact, the extent of persulfidation of proteins is increased significantly in plants growing under conditions of suppressed photorespiration in comparison with control plants. Specifically, 1096 persulfidated proteins were identified in Arabidopsis plants grown under NPC (García-Calderón *et al*., 2023), of which 98.7% showed higher levels of persulfidation under NPC than under control conditions. The identified proteins were mainly involved in central metabolic pathways such as glycolysis, the tricarboxylic acid (TCA) cycle, redox homeostasis, and the pentose phosphate pathway, as well as nitrogen and sulfur assimilation, highlighting the broad regulatory scope of H_2_S. Such persulfidated protein enrichment postulates H_2_S as a fundamental regulator of redox state and metabolic adaptation under conditions of elevated carbon availability and altered nitrogen cycling.

Under conditions of suppressed photorespiration, plants experience a limitation of NADH, which is essential for nitrate reduction. This negatively affects nitrogen assimilation and disrupts amino acid biosynthesis. In particular, in Arabidopsis, excessive carbon accumulation and decreased nitrogen assimilation occur, resulting in a significant imbalance in the carbon/nitrogen (C/N) ratio (García-Calderón *et al*., 2023). To counteract these effects, exogenous H_2_S treatment is able to reactivate nitrogen assimilation pathways, likely through persulfidation of key enzymes such as glutamine synthetase (GS) and glutamate synthase (GOGAT), probably by improving their catalytic activity and stability conditions. In fact, several studies have demosntrated that treatment with exogenous H_2_S increases the activities of several nitrogen-metabolism proteins, such as nitrate reductase (NR), glutamate dehydrogenase (GDH), nitrite reductase (NiR), GS, and GOGAT (Kaya and Shabala, 2023; Raju and Prasad, 2023; Kharbech *et al*., 2024; Zhao *et al*., 2024). These changes probably contribute to re-establishing the C/N balance and recovering the levels of amino acids affected in such conditions (García-Calderón *et al*., 2023).

In addition to all of the above, high CO_2_ concentrations suppress photorespiration, which alters energy metabolism. Under these conditions, plants exhibit a marked decrease in ATP content, reflecting mitochondrial dysfunction and altered carbon metabolism. H_2_S has been shown to partially restore ATP content, indicating that it has an important role in maintaining cellular homeostasis from an energetic perspective. It is likely that this effect involves the persulfidation of key enzymes that participate in processes such as glycolysis, the TCA cycle, and mitochondrial respiration. Taken together, these findings indicate that H_2_S contributes to maintaining energy balance by reprogramming core metabolic pathways under carbon-rich and stress-mimicking conditions (Aroca *et al*., 2023; García-Calderón *et al*., 2023).

In line with these observations, it was demonstrated that exogenous H_2_S enhances photosynthesis in *Spinacia oleracea* (Chen *et al*., 2011). Interestingly, this work showed that genes encoding the large and small subunits of Rubisco (RBCL, RBCS), ferredoxin/thioredoxin system components (FTR, FRX, TRX-m, TRX-f), and NADP-malate dehydrogenase (NADP-MDH) were up-regulated, whereas genes involved in photorespiration, such as glycolate oxidase (GYX) and cytochrome oxidase (CCO), were down-regulated after H_2_S treatment. These results suggest that, beyond its effects under NPC, H_2_S can directly enhance photosynthetic performance and reduce losses from photorespiration, thus offering a dual protective role by improving carbon assimilation efficiency while alleviating oxidative stress and metabolic disturbances (Chen *et al*., 2011).

In summary, elevated CO_2_ increases carbon fixation, but causes alterations in photorespiration, nitrate assimilation, and redox balance. In this context, H_2_S and its associated persulfidation mechanism emerge as vital compensatory systems (Aroca *et al*., 2023; García-Calderón *et al*., 2023). H_2_S acts as a modulator of redox homeostasis that can be supported by the increase of cysteine and GSH levels under H_2_S treatment. It also helps to restore the C/N balance as well as the energy balance when photorespiration is limited. Therefore, H_2_S plays a key role in general stress responses (Aroca *et al*., 2021; Zhang *et al*., 2021). All this suggests that H_2_S signaling pathways could be a potentially useful approach to preserve crop yields and quality under future climatic conditions, including the ongoing increase of atmospheric CO_2_ (Aroca *et al*., 2023).

### Drought

Drought is recognized as the most severe environmental constraint, causing greater yield losses than any other abiotic stress and severely impacting crop production worldwide (Rojas, 2020). To survive, plants possess the ability to cope with drought stress through a variety of strategies, including biochemical mechanisms and physiological adjustments (Gupta *et al*., 2020; Kim *et al*., 2024). Dehydration triggers the synthesis of the phytohormone abscisic acid (ABA), which promotes stomatal closure to minimize water loss, and activates the expression of genes involved in signal transduction pathways (Cutler *et al*., 2010). In addition, stomatal closure under drought conditions impacts energy metabolism by compromising photosynthetic efficiency and altering growth-related programs (H. Zhang *et al*., 2020). Drought stress also leads to the accumulation of ROS, resulting in the disruption of redox homeostasis and, in consequence, prompting the activation of the antioxidant defense systems (Miller *et al*., 2010).

Regardless of the activation of inherent defense mechanisms, the severity of the stress often exceeds the plant’s capacity for tolerance, highlighting the need for the development of additional protective strategies. A substantial body of research has demonstrated the key role of H_2_S in improving plant tolerance to drought stress by modulating and strengthening plant response mechanisms. Through this work, significant insight into the regulation by H_2_S of ABA-dependent stomatal closure has been gained. From the initial report proposing H_2_S as a novel component of ABA signaling in guard cells (Scuffi *et al*., 2014), research mainly in Arabidopsis has elucidated the molecular mechanism of H_2_S action leading to ABA-dependent stomatal closure, through the persulfidation of proteins. The well-studied LCDES from Arabidopsis, DES1, is activated by the increase of ABA under drought, inducing the production of H_2_S to persulfidate specific cysteines on proteins involved in the stomatal movement, such as open stomata 1 (OST1) (Chen *et al*., 2021), slow anion channel current 1 (SLAC1) (Wang *et al*., 2016), the NADPH oxidase RBOHD (respiratory burst oxidase homolog D) (Shen *et al*., 2020), the transcription factor ABI4 (Zhou *et al*., 2021), and the KAT1 subunit of inward potassium channels (Liu *et al*., 2025). A recent review highlighted the complex network of interactions between H_2_S and signaling molecules such as ABA, H_2_O_2_, NO, CO, melatonin, and Ca^2+^ in the regulation of stomatal movement (Jin *et al*., 2025 ).

One of the primary contributions of H_2_S to enhance drought tolerance is the modulation of ROS levels and its ability to balance oxidative stress. Through various approaches, such as exogenous H_2_S application combined with drought stress, pretreatment with H_2_S prior to imposing drought stress, or analyzing endogenous H_2_S production, a substantial number of studies has demonstrated a clear reduction in ROS content and regulation of antioxidative defenses, through either antioxidant enzymes or antioxidant metabolites, mediated by H_2_S (see, for example, Shan *et al*., 2014; Zhou *et al*., 2020; Amir *et al*., 2021; Jurado-Flores *et al*., 2023a; Wang *et al*., 2024; Zhang *et al*., 2024; Carrillo *et al*., 2025).

H_2_S also improves drought tolerance by regulating the metabolic reprogramming that occurs to sustain plant growth and survival. In response to water limitation, alterations in amino acid metabolism are made for the synthesis of stress-induced proteins and secondary metabolites, and the accumulation of proline as a major osmolyte (Batista-Silva *et al*., 2019). Furthermore, drought hampers the functioning of photosynthesis, leading to a deficiency of energy, the depletion of carbohydrates, and the utilization of fatty acids and amino acids for energy production (Heinemann and Hildebrandt, 2021). Thereby, a significant decrease in the amino acids accumulated upon drought was observed when H_2_S was applied to Arabidopsis and barley plants (Jurado-Flores *et al*., 2023a; Carrillo *et al*., 2025). Photosynthetic performance was also enhanced in tobacco under drought stress by the addition of H_2_S, mainly through maintaining the activity of photosystem II (Wang *et al*., 2024). Furthermore, H_2_S regulates energy production under drought in Arabidopsis and rice (Jin *et al*., 2018; Zhang *et al*., 2024).

Another relevant strategy through which H_2_S enhances plant drought resistance is the regulation of water transport activity connected to aquaporins, as recently demonstrated in rice protoplasts (Zhang *et al*., 2024). H_2_S was found to inhibit protoplast water permeability, thereby mitigating water loss during drought stress in plants characterized by high transpiration and greater sensitivity to water deficit.

Recent studies in three experimental models, barley (*Hordeum vulgare*) (Carrillo *et al*., 2025), *Arabidopsis thaliana* (Jurado-Flores *et al*., 2023a), and rice (*Oryza sativa*) (Zhang *et al*., 2024), demonstrate that H_2_S acts primarily through protein persulfidation to improve drought tolerance. In this regard, higher levels of persulfidation were detected under drought stress when barley plants were pretreated with H_2_S, and correlated with enhanced stress tolerance (Carrillo *et al*., 2025). In Arabidopsis, quantitative proteomic analysis identified 887 proteins exhibiting significantly altered persulfidation levels when compared with control and drought-stressed plants. Notably, proteins with increased persulfidation levels in drought-stressed plants were predominantly involved in the response to oxidative stress and H_2_O_2_ catabolism. This observation suggests that H_2_S-dependent persulfidation plays a crucial role in maintaining redox homeostasis under drought conditions (Jurado-Flores *et al*., 2023a). In rice, a similar proteomic approach identified 843 proteins with differential levels of persulfidation. The proteins showing higher levels of persulfidation under drought stress were involved in the mitigation of oxidative stress, the TCA cycle, and energy processes. Proteins involved in ion and water transport were also identified, with the aquaporin family showing the highest differential levels of persulfidation. The most relevant finding was the increase in aquaporin activity mediated by persulfidation in rice, which favored water transport efficiency and improved water retention under drought conditions. This specific regulation was not observed in Arabidopsis, highlighting the interspecific variability in the response to H_2_S (Zhang *et al*., 2024).

An outstanding finding of the above-described studies in three experimental models is that H_2_S reduces the harmful impact of drought when plants are pretreated before being subjected to the stress condition. Therefore, the role of H_2_S is to prepare the plant to respond to subsequent drought conditions in a more efficient manner, a process that resembles priming, which has been previously described for a variety of agents (Antoniou *et al*., 2016; Mauch-Mani *et al*., 2017; Tripathi *et al*., 2024). These findings warrant further investigation as they will contribute to the development of agricultural strategies to improve plant resilience in the face of stress.

### Salinity

At present, around 1125 million hectares of land are affected by salt, with about 76 million hectares being impacted by salinization and sodification due to anthropogenic activities (Hossain, 2019). H_2_S has been shown to play a critical role in mitigating the adverse effects of salinity stress in plants, with several key mechanisms contributing to its protective effects.

Salinity stress induces the endogenous production of H_2_S in crop plants; a 2- to 4-fold increase in endogenous H_2_S production compared with control in response to salinity stress was reported in *Cynodon dactylon* (Shi *et al*., 2013) and, similarly, in *Medicago sativa* due to salinity-induced LCDES activity (Lai *et al*., 2014). This H_2_S induction in turn enhances salinity tolerance by modulating the activity of antioxidant enzymes, increasing the osmolyte content, regulating cell signaling proteins, maintaining ionic homeostasis, and regulating the expression of stress-linked genes (Zulfiqar and Hancock, 2020). It was previously found that the exogenous application of H_2_S donors enhanced osmolyte production and the activity of osmolyte biosynthesis enzymes (Khan *et al*., 2018; Pandey and Gautam, 2020).

Additionally, the ameliorative impact of exogenous H_2_S supply on salinity-induced stress in plants encompasses the enhancement of seed germination, ionic equilibrium, strengthening of photosynthetic attributes, reinforcement of the antioxidant defense system, and induction of osmoregulatory processes (Goyal *et al*., 2021). During seed germination in *Cucumis sativus* and *M. sativa*, the application of exogenous H_2_S promoted starch breakdown and increased α- and β-amylase activity, supporting seedling growth (Wang *et al*., 2012; Lai *et al*., 2014).

Regarding ionic equilibrium, the effect of H_2_S on modulating the Na⁺/K⁺ balance has been observed in multiple plant species. In rice, inhibition of Na⁺ uptake by H_2_S helps constrain the Na⁺/K⁺ ratio, thereby preserving plasma membrane integrity under salinity stress (Mostofa *et al*., 2015a). In barley, H_2_S treatment induced the expression of genes encoding inward-rectifying potassium channels (HvAKT1) and high-affinity K⁺ transport systems (HvHAK4) (Chen *et al*., 2015). In addition, in barley, pretreatment with NaHS resulted in a suitable Na⁺/K⁺ ratio by increasing K⁺ levels, likely due to the persulfidation of ion channels such as KAB1, which is involved in K⁺ uptake (Carrillo *et al*., 2025). In Arabidopsis, the persulfidation of the voltage-gated potassium channel subunit beta was identified in a proteomic study (Jurado-Flores *et al*., 2021), and, very recently, the specific cysteine residues that are persulfidated in the KAT1 subunit of inward potassium channels have been identified (Liu *et al*., 2025). These findings highlight the crucial role of protein persulfidation in enhancing plant tolerance to salinity stress by optimizing ion balance and improving cellular function. At the molecular level, H_2_S also regulates the expression of the SOS pathway, which is essential for maintaining Na⁺ and K⁺ homeostasis under salt stress. Thus, in Arabidopsis, H_2_S treatment increased the expression of genes such as *SOS2*, *SOS3*, and *SOS4* (Christou *et al*., 2013).

Furthermore, H_2_S has been shown to enhance photosynthetic efficiency in salt-stressed strawberry (*Fragaria × ananassa*) and rice, increasing the chlorophyll and carotenoid contents while improving stomatal conductance (Christou *et al*., 2013; Mostofa *et al*., 2015b). H_2_S has been also proved to enhance salt tolerance in rice by mitigating oxidative stress, recovering photosynthetic capacity, improving primary and energy metabolism, and strengthening protein metabolism. A recent proteomic analysis described that its protective effect against salt stress was linked to key proteins involved in photosynthesis (PsbQ domain protein, plastocyanin oxidoreductase iron–sulfur protein), the Calvin cycle (phosphoglycerate kinase, sedoheptulose-1,7-bisphosphatase precursor, ribulose-1,5-bisphosphate carboxylase/oxygenase), and chlorophyll biosynthesis (glutamate-1-semialdehyde 2,1-aminomutase, coproporphyrinogen III oxidase). Additionally, NaHS up-regulated ATP synthesis-related proteins, malate dehydrogenase, and 2,3-bisphosphoglycerate-independent phosphoglycerate mutase (Wei *et al*., 2021).

H_2_S bolsters the antioxidant defense system under salt stress by increasing the activity of key enzymes such as SOD and peroxidase in *C. sativus*, and by stimulating enzymes required for the biosynthesis of non-enzymatic antioxidants, such as glutathione and ascorbic acid, in maize (*Zea mays*) (Yu *et al*., 2013; Shan *et al*., 2014). Furthermore, it improves oxidative stress tolerance by enhancing glyoxalase I and II activities to regulate methylglyoxal content in *O. sativa* and *Brassica oleracea* (Mostofa *et al*., 2015b; Montesinos-Pereira *et al*., 2018), and by decreasing the activity of lipoxygenase, which is linked to lipid peroxide synthesis (Wang *et al*., 2012; Christou *et al*., 2013; Shi *et al*., 2013; Lai *et al*., 2014). All these data show the potential of H_2_S for alleviating oxidative stress in various plant species.

Finally, in relation to its role as an osmoregulatory agent, the exogenous application of H_2_S led to an accumulation of sucrose, proline, and other soluble sugars in *Cynodon dactylon* subjected to NaCl-induced stress (Shi *et al*., 2013). Collectively, these multifaceted effects make H_2_S a key player in improving plant resilience to salinity stress.

### Thermal stress

The increasing global average temperature, by about 0.85 °C over the past century, is a major abiotic stress that severely restrains crop growth, development, and yield, endangering global food security. Plants’ endogenous mechanisms are often insufficient to cope with rapid environmental changes, and therefore a full understanding of the regulatory mechanisms controlling plant temperature tolerance is essential for developing strategies to improve crop resilience.

High-temperature stress triggers a cascade of detrimental physiological and biochemical changes in plants, including oxidative damage from the overproduction of ROS, impaired photosynthetic efficiency, and loss of membrane integrity. The exogenous application of H_2_S has been shown to enhance thermotolerance in several species, from Arabidopsis to major crops such as maize and strawberry (Ye *et al*., 2020; Alvi *et al*., 2024a; Z.-G. Li *et al*., 2024). Additionally, H_2_S endogenously generated by DES1 has been demonstrated to enhance plant basal thermotolerance (Fang *et al*., 2025).

As stated for other abiotic stresses, H_2_S dynamically boosts the repertoire of the cellular antioxidant system by increasing the expression and activity of the key antioxidant enzymes (Zhou *et al*., 2018; Aroca *et al*., 2020). By enhancing this enzymatic barrier, H_2_S can efficiently attenuate the ROS burst caused by heat stress, which in turn prevents lipid peroxidation and protects the integrity of cellular membrane. The protective action of H_2_S functions mainly via the ascorbate–glutathione cycle (Liu *et al*., 2020). This cycle is important for scavenging ROS, and H_2_S up-regulates the activity of its key antioxidant enzymes, such as APX1 and glutathione reductase 1 (GR1), by persulfidation, increasing their activity. Likewise, CAT is persulfidated, provoking an important modification of its activity in H_2_O_2_ detoxification (Li *et al*., 2014; Aroca *et al*., 2015; Ye *et al*., 2020; García-Calderón *et al*., 2023; De Brasi-Velasco *et al*., 2025). On the other hand, the major enzyme controlling ROS levels in the apoplast in response to stress is the NADPH oxidase RBOHD, which is also controlled by persulfidation on specific cysteine residues (Shen *et al*., 2020), leading to dampening of the oxidase activity, thus providing a negative feedback loop to prevent the accumulation of excess ROS, which can be damaging. Therefore, H_2_S shows a dual role in regulating the production and detoxification of ROS.

The induction of heat shock proteins (HSPs) is also an important part of thermotolerance. During temperature stress, plants respond by activating the heat stress response, to more effectively survive hot temperatures. An essential step during this response is the induction of HSPs, which exert a chaperone function and are involved in refolding damaged proteins to maintain cellular integrity and homeostasis by preventing aggregation. H_2_S has been shown to promote the expression of HSPs, notably HSP90 and HSP70, providing a direct mechanism for maintaining proteostasis under heat stress (Min *et al*., 2016). In fact, several HSP90 and HSP70 proteins have also been found to be targets of persulfidation (Aroca *et al*., 2017; Laureano-Marin *et al*., 2020; Jurado-Flores *et al*., 2023b).

The signaling network of H_2_S also involves crosstalk with other key stress hormones such as ABA, as described earlier. This hormone is a central regulator of abiotic stress responses, to fine-tune stomatal closure and water balance during heat events (Laureano-Marin *et al*., 2020; J. Zhang *et al*., 2020; Wang *et al*., 2023). In fact, it has been described that H_2_S and ABA mutually enhance their internal levels in maize seedlings, playing an essential role in conferring heat tolerance by activating the ROS-scavenging system (Wang *et al*., 2023). Under heat stress, H_2_S generated by DES1 induces stomatal closure by regulating the expression of K^+^ channel-encoding genes, such as *KAT1* and *KC1*, thus preventing excess water loss. H_2_S also enhances the stomatal density by increasing the transcriptional levels of stomatal development-associated genes (Fang *et al*., 2025). Besides, H_2_S-mediated persulfidation of ABI4 enhances its stability and ability to bind to DNA during heat stress, allowing it to transactivate downstream targets such as MAPKKK18, thereby integrating the H_2_S and ABA pathways to fine-tune the stress response (Zhou *et al*., 2021). Therefore, the modification of key transcriptional regulators allows for broad changes in stress-responsive gene expression related to temperature stress.

Regarding cold temperature, H_2_S has also emerged as a vital component in plant acclimation to cold. Low temperatures, including chilling (0–15 °C) and freezing (<0 °C) conditions, disrupt metabolic activity, induce membrane rigidification, and cause cellular dehydration. H_2_S application alleviates chilling injury in sensitive crops such as banana, cucumber, tomato, and grape (Nasibi *et al*., 2020; Wu *et al*., 2024). In addition to redox signaling, H_2_S regulates other key cold-response pathways. By controlling Ca^2+^ channel and pump activities, it modulates Ca^2+^ signaling, a rapid and widespread response to chilling, and contributes to the maintenance of ionic stability. In addition, H_2_S interacts with the C-repeat Binding Factor (CBF) signaling pathway, a master regulating cascade of cold acclimation. H_2_S may initiate the expression of an entire battery of *COLD-REGULATED GENES* by modulating the expression or activity of CBF transcription factors, the protein products of which contribute to freezing tolerance by the protection of membranes and the reduction of dehydration (Guo *et al*., 2025).

In addition, a significant number of proteins involved in signaling and metabolism have been identified to undergo persulfidation under both high- and low-temperature stress (Du *et al*., 2021; Z.-G. Li *et al*., 2024 ). Studies have shown that heat stress significantly increases endogenous H_2_S production (Li *et al*., 2015). This surge in H_2_S is not merely a stress by-product but a crucial signaling event. Elevated H_2_S levels then lead to the persulfidation of a wide array of proteins, including key regulators of the heat stress response itself and transcription factors, thereby H_2_S is fine-tuning the cell’s ability to cope with thermal damage.

## Conclusion

H_2_S signaling has emerged in recent years as a highly relevant topic. Particularly in plants and within the context of climate change, it has been shown that H_2_S regulates plant responses to adverse environmental conditions and promotes plant resilience. Therefore, H_2_S exhibits significant potential in enabling plants to counteract the harmful effects of climate change.

H_2_S regulates essential processes that are commonly affected by a variety of stresses, such as cellular redox homeostasis, by reducing ROS levels. It also promotes metabolic reprogramming in response to impaired photosynthesis, thereby enabling ATP production. Additionally, it modulates secondary metabolism for the synthesis of defense-related compounds and also regulates hormonal signaling (Fig. 4). Moreover, H_2_S also regulates more specific responses to particular stress conditions, such as ionic balance under salt stress. Although current knowledge remains limited, several studies, primarily focused on drought stress, have highlighted protein persulfidation as the main mechanism through which H_2_S exerts its signaling function.

**Fig. 4.**
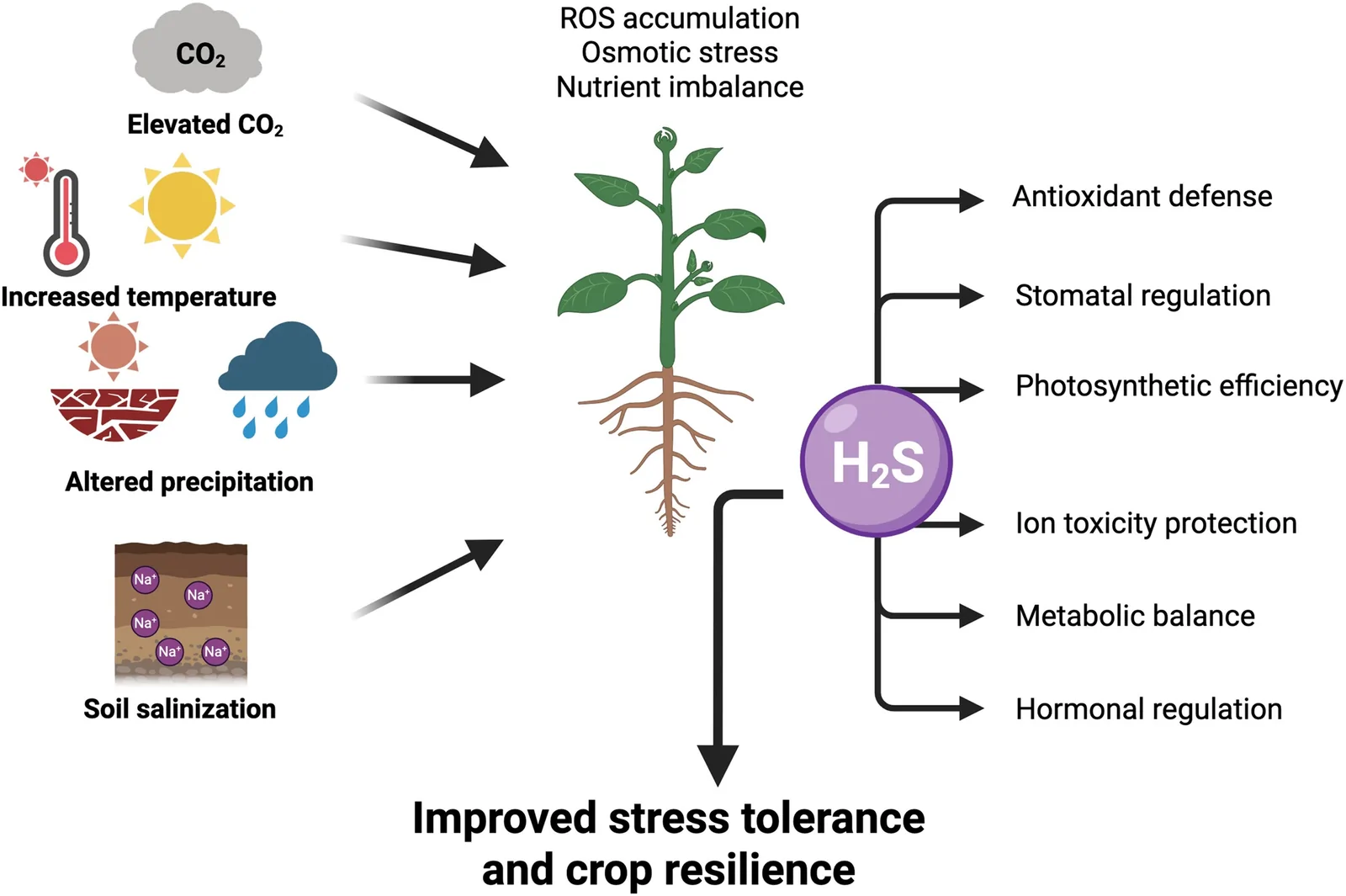
Hydrogen sulfide (H_2_S) regulates plant physiological responses to environmental stressors associated with climate change. Abiotic factors such as elevated atmospheric carbon dioxide (CO_2_,) rising temperatures, altered precipitation patterns, and soil salinization disrupt plant homeostasis, leading to reactive oxygen species (ROS) accumulation, osmotic stress, and nutrient imbalances. H_2_S modulates a suite of adaptive mechanisms, including stomatal regulation, antioxidant defense systems, enhanced photosynthetic efficiency, ion toxicity mitigation, metabolic reprogramming, and hormonal signaling pathways that collectively improve stress tolerance and promote crop resilience under changing climatic conditions. Created in BioRender. Romero, L. (2026) https://BioRender.com/gt1duf4.

From an agronomic perspective, the possibility of applying pretreatments with H_2_S donors could represent an effective strategy for physiologically fortifying plants before they are exposed to periods of stress. In crops of high economic value, such as barley and rice, this strategy could reduce yield losses and maintain grain quality under stress conditions.
